# Two TIR-like domain containing proteins in a newly emerging zoonotic *Staphylococcus aureus* strain sequence type 398 are potential virulence factors by impacting on the host innate immune response

**DOI:** 10.3389/fmicb.2014.00662

**Published:** 2014-12-09

**Authors:** Nicholas J. Patterson, Juliane Günther, Amanda J. Gibson, Victoria Offord, Tracey J. Coffey, Gary Splitter, Ian Monk, Hans-Martin Seyfert, Dirk Werling

**Affiliations:** ^1^Molecular Immunology Group, Department of Pathology and Pathogen Biology, Royal Veterinary CollegeHatfield, UK; ^2^Leibniz Institute for Farm Animal BiologyDummerstorf, Germany; ^3^School of Veterinary Medicine and Sciences, Faculty of Medicine and Health Sciences, University of NottinghamSutton Bonington, UK; ^4^Department of Pathobiological Sciences, University of Wisconsin–MadisonMadison, WI, USA; ^5^Department of Microbiology and Immunology, Faculty of Medicine, Dentistry and Health Sciences, University of MelbourneMelbourne, VIC, Australia

**Keywords:** TLR signaling, *Staphylococcus aureus*, bacterial proteins, TCP, innate immune response, mouse model

## Abstract

*Staphylococcus aureus*, sequence type (ST) 398, is an emerging pathogen and the leading cause of livestock-associated methicillin-resistant *S. aureus* infections in Europe and North America. This strain is characterized by high promiscuity in terms of host-species and also lacks several traditional *S. aureus* virulence factors. This does not, however, explain the apparent ease with which it crosses species-barriers. Recently, TIR-domain containing proteins (Tcps) which inhibit the innate immune response were identified in some Gram-negative bacteria. Here we report the presence of two proteins, *S. aureus* TIR-like Protein 1 (SaTlp1) and *S. aureus* TIR-like Protein 2 (SaTlp2), expressed by ST398 which contain domain of unknown function 1863 (DUF1863), similar to the Toll/IL-1 receptor (TIR) domain. In contrast to the Tcps in Gram-negative bacteria, our data suggest that SaTlp1 and SaTlp2 increase activation of the transcription factor NF-κB as well as downstream pro-inflammatory cytokines and immune effectors. To assess the role of both proteins as potential virulence factors knock-out mutants were created. These showed a slightly enhanced survival rate in a murine infectious model compared to the wild-type strain at one dose. Our data suggest that both proteins may act as factors contributing to the enhanced ability of ST398 to cross species-barriers.

## INTRODUCTION

*Staphylococcus aureus* is an important commensal and pathogen of animal species causing a variety of diseases ranging from skin and soft tissue infections to more severe, life threatening infections such as toxic shock syndrome in both human and animal species (Lowy, 1998). In addition, *S. aureus* is a major pathogen causing bovine mastitis, a disease of high economic importance (Holmes and Zadoks, 2011). Here, infections with *S. aureus* mainly causes subclinical infections of the udder parenchyma, which is accompanied by the absent of the expression of early innate immune defense genes when compared to infection with *Escherichia coli* (Petzl et al., 2008). Indeed, comparative kinetic analysis between *E. coli* and *S. aureus* infected primary bovine mammary epithelial cells (pbMECs) showed that *S. aureus* infection failed to induce tumor necrosis factor (TNF), interleukin (IL)-1 and IL-8 (CXCL8), which did not depend on the lack of recognition by bovine Toll-like receptor (TLR) 2 or 4 (Yang et al., 2008; Gunther et al., 2011). Treatment of *S. aureus* infections is complicated by its propensity to acquire antibiotic resistance determinants, most notably the emergence of methicillin-resistant *S. aureus* (MRSA). Human MRSA infections can be classified as being community-associated (CA-MRSA), healthcare-associated (HA-MRSA), or livestock-associated (LA-MRSA; Lindsay, 2010). Although most *S. aureus* strains are considered to be host specific (Fitzgerald, 2012), interest in livestock-associated *S. aureus* was renewed with the discovery of MRSA sequence type (ST) 398, initially in pigs and more recently in calves, chickens, horses, and pets (Armand-Lefevre et al., 2005; Graveland et al., 2011). ST398, as determined by multilocus sequence typing (MLST), is considered a newly emerging, pathogenic and zoonotic strain and the major cause of LA-MRSA in Europe and North America (Smith et al., 2009; Graveland et al., 2010), displaying significant diversity and high content of antimicrobial resistance genes, but so far, no significant virulence genes compared to other ST-lineages have been identified (Jamrozy et al., 2012). Current data indicates a low incidence of human to human transmission of this strain suggesting that the majority of infections are of a zoonotic origin (Wassenberg et al., 2011). Interestingly, it has been shown that ST398 can be either MRSA or methicillin-sensitive *S. aureus* (MSSA), both having been isolated from infections of a range of domestic animals including pigs, horses, chickens, and cows (Witte et al., 2007). Additionally, an increase in the incidence rate of clinical mastitis cases due to ST398 has been reported in recent years, adding to the zoonotic risk (Holmes and Zadoks, 2011). In this regard, it is also worth mentioning that infection of the udder by *S. aureus* does not seem to induce an appropriate innate immune response (Yang et al., 2008), potentially indicating some immune evasion strategies. Given these observations, the relative ease with which ST398 crosses the species-barrier and the lack of certain important *S. aureus* virulence factors such as panton-valentine leukocidin (PVL), toxic shock syndrome toxin-1 (TSST-1), and leukotoxin M (LukM; Vanderhaeghen et al., 2010) it is possible that ST398 may possesses additional and non-traditional virulence factors. One such group of virulence factors could be represented by Toll/IL-1 receptor (TIR)-domain containing proteins (Tcps), which have been identified in various bacteria (Spear et al., 2009). Whereas Tcps may have a normal function in protein–protein interaction (Spear et al., 2009), the presence of some Tcps in Gram-negative bacteria has been shown to shut-down TLR signaling (Spear et al., 2009). TLRs are vital components of the innate immune system, recognizing microbe-associated molecular patterns (MAMPs), leading to activation of transcription factors important for initiating the immune response (Akira et al., 2006). The intracellular TIR domain of TLRs is responsible for recruitment and activation of TIR-domain containing adaptor molecules such as myeloid differentiation factor 88 (MyD88), TIR-domain containing adaptor inducing interferon-β (TRIF), TIR-domain containing adaptor protein (TIRAP), and sterile-α and HEAT/Armadillo motifs-containing protein (SARM; McGettrick and O’Neill, 2004). Tcps share low sequence identity to their mammalian counterparts, and both are characterized by the presence of three boxes of conserved residues set in a core sequence ranging from 135 to 160 amino acids (Rana et al., 2011). In addition to Tcps, several additional protein classes have recently been identified that may interact and interfere with TLR signaling. A conserved region has been identified in transmembrane receptors including similar expression to fibroblast-growth factor (SEF) and IL17Rs in eukaryotes and bacteria. This region shows structural homology to TIR domains, the key functional subdomain used by the Toll/IL-1 family receptors. A similar domain is also found in Act1/CIKS, an essential adaptor downstream of IL-17R family members that is required for activation of NF-κB and other signals. Thus, the SEF/IL17R/CIKS/Act1 homology (SEFIR) domain is observed in both transmembrane and cytoplasmic proteins (Onishi et al., 2010). Sequence and structure-based fold prediction server results support the distant similarity of the SEFIR domain to the TIR structure, except for the most C-terminal region. SEFIR and TIR domains are related, belonging to a new STIR domain superfamily consisting of SEFIR, TIR and the recently identified TIR-like domain of unknown function (DUF) 1863 (Novatchkova et al., 2003). In Toll/IL-1R-like pathways, the cytoplasmically localized TIR domain of a receptor and the TIR domain of a soluble adaptor interact physically and activate signaling. The similarity between the SEFIR and TIR domains involves the above mentioned conserved boxes 1 and 2 of the TIR domain that are implicated in homotypic dimerization, but there is no sequence similarity between SEFIR domains and the TIR sequence box 3. By analogy, it was suggested that SEFIR-domain proteins function as signaling components of Toll/IL-1R-similar pathways and that their SEFIR domain mediates physical protein–protein interactions between pathway components (Wu et al., 2012).

Several studies have revealed that a diverse range of both pathogenic and non-pathogenic microorganisms express proteins containing STIR domains (Newman et al., 2006; Cirl et al., 2008; Spear et al., 2012). Despite low sequence identity, the crystal structure of *Paracoccus denitrificans* (PdTIR) is structurally similar to the TIR domain of human TLR1. PdTIR interacts with both MyD88 and the TLR4 TIR domains, however, no inhibitory function has been demonstrated (Low et al., 2007; Chan et al., 2009). In contrast, only Tcps identified in Gram-negative bacteria have been shown to inhibit MyD88-dependent TLR signaling. TlpA from *Salmonella enterica* inhibits activation of the transcription factor NF-κB (Newman et al., 2006). Similarly TcpB, present in a range of *Brucella* species including *B. melitensis, B. ovis* and *B. abortus,* and TcpC from *E. coli* inhibits NF-κB activation by direct interaction with MyD88, subsequently aiding bacterial macrophage entry and enhancing degradation of MAL (Radhakrishnan et al., 2009, 2011; Radhakrishnan and Splitter, 2010). In contrast, the *Yersinia pestis* TIR-domain protein (YpTdp) has been shown to interact with MyD88 and down-regulate activation of IL-1β; although the presence of this protein did not affect the phenotype of deletion mutants in a mouse infection model (Rana et al., 2011; Spear et al., 2012). There is less information available regarding SEFIR- (Wu et al., 2012) and DUF1863-domain containing proteins within the STIR superfamily.

The hypothesis of the current work was to assess whether proteins belonging to these families are in general present in Gram-positive bacteria, and specifically in ST398, which may explain the ease with that this specific *S. aureus* crosses into other species. Here we present data identifying two DUF1863-domain containing proteins, named *S. aureus* TIR-Like Proteins 1 and 2 (SaTlp1 and SaTlp2) in ST398 *S. aureus*, which affect TLR-dependent NF-κB activation in HEK293 and pbMECs. Initial experiments showed that a knock-out (KO) mutation of ST398 lacking both proteins seemed to enhanced survival of mice in an *in vivo* infection model using a defined colony forming unit (CFU), although the data did not reach a level of significance. These proteins may be important virulence factors and contribute to the ability of ST398 to cross species-barriers.

## MATERIALS AND METHODS

### CELLS CULTURE CONDITIONS

HEK293 cells do not express TLR2 or TLR5 on their own, and do not respond to the appropriate TLR ligands (Willcocks et al., 2013; Metcalfe et al., 2014). HEK293 cells stably transfected with bovine TLR2 (GenBAnk Acc. No AY634629; HEK-boTLR2) were generated in house (Willcocks et al., 2013). HEK cells expressing human TLR5 were purchased from Invivogen (293-hTLR5), and have been used before (Metcalfe et al., 2014). Expression of TLRs was maintained using Geneticin (Gibco) at 1 mg ml^-1^ for boTLR2 and Blasticidin (Lifetech) at 10 μg ml^-1^ for 293-hTLR5. Both cell types were grown in DMEM (Gibco) supplemented with 10% fetal calf serum (PAA). The preparation of pbMECs as cells that have been shown to be affected by *S. aureus* expression, their culture in RPMI 1640 (Gibco) on collagen-coated plates, and general challenge conditions were previously described (Gunther et al., 2011).

### BACTERIAL STRAINS, GROWTH CONDITIONS, AND MLST

*S. aureus* strains isolated from various species and disease presentations (**Table 1**), were grown on 5% Sheep blood agar (Oxoid) and cultured in brain heart infusion medium (Oxoid) at 37°C. Bacterial genomic DNA was isolated using a Wizard^®^ Genomic DNA Purification Kit (Promega) following manufacturer’s guidelines. Polymerase chain reaction (PCR) was performed with Easy-A High-Fidelity PCR Mastermix (Agilent) using the primers stated on http://saureus.mlst.net/ (Aanensen and Spratt, 2005) for the genes *arcC*, *aroE*, *glpF*, *gmk*, *pta*, *tpi,* and *tpi*. Each primer pair amplified an internal fragment of the housekeeping gene and allowed accurate sequencing of ∼450-bp fragments of each gene on both strands. For each locus, the sequences obtained from all isolates were compared and the different sequences were assigned allele numbers. For each isolate, the alleles at each of the seven loci defined the allelic profile that corresponded to a ST.

**Table 1:** Names, species isolated from, MLST group, methicillin-sensitivity (when relevant) and presence of SaTlp1 of the *Staphylococcus aureus* strains.

Isolate	Source	ST	Methicillin	SaTlp1 ±
5x	Bovine	398	MRSA	±
6x	Bovine	398	MRSA	±
HPA34809	Equine	398	MRSA	±
HPA35009	Equine	398	MRSA	±
HPA35209	Equine	398	MRSA	±
Pil78	Porcine	398	MSSA	±
Pil79	Porcine	398	MSSA	±
Pil80	Human	398	MSSA	±
Pil 82	Porcine	398	MSSA	±
Pil 83	Human	398	MSSA	±
4x	Bovine	1786		-
1027	Bovine	133		-
177/10	Bovine	97		-
465/07	Bovine	504		-
567/07	Bovine	97		-
642/07	Bovine	1380		-
A94	Canine	CC22 or CC30/ST36	MRSA	-
A95	Canine	CC22 or CC30/ST36	MRSA	-
B019	Canine	CC22 or CC30/ST36	MSSA	-
B020	Canine	CC22 or CC30/ST36	MSSA	-

### IDENTIFICATION AND CLONING OF BACTERIAL Tcps

Identification of Tcp-like proteins within *S. aureus* sequences were performed by searching for homologs of mammalian and bacterial STIR domains in the non-redundant protein sequences database^1^; using the PSI-BLAST algorithm (Altschul et al., 1997). Identified proteins were screened initially for structural similarities to known Tcp using SMART (Schultz et al., 1998^2^) and the similarity of their tertiary structure compared to other proteins using the BioInfoBank Meta Server^3^. Subsequently, primers (**Table 2**) were used to amplify the sequences of *SaTlp1* and *SaTlp2* (GenBank Acc. No. CAQ50581.1 and CAQ50581.2, respectively) which also introduced a mutation to alter the start codons to ATG, allowing transcription in eukaryotic cells. PCR products were excised from 1% agarose gels using a GenElute Gel Extraction Kit (Sigma), cloned into pGEM-T Easy (Promega) and inserts sequenced. Subsequently, *SaTlp1, SaTlp2,* and *TcpB* were cloned into the pcDNA3.3 TOPO plasmid (Invitrogen). All plasmids were propagated in TOP10 *E. coli* (Invitrogen) bacteria, grown in Luria Broth supplemented with 100 μg ml^-1^ ampicillin (Invivogen), purified using a PureYield Plasmid Miniprep kit (Promega) and subsequently used for transfection into HEK-boTLR2, 293-hTLR5, or pbMEC cells.

**Table 2:** Primers used for cloning and subsequent screening for *SaTlp1* and *SaTlp2*, and subsequent cloning into pcDNA3.3 and pIMAY.

Primer	Sequence
**pcDNA3.3 cloning**
SaTlp1-5′+Kozak	GCG ATG GAA AGA CAA CAA AC
SaTlp1-3′	CTA ATC TGA AAA AGC CTC
SaTlp2-5′+Kozak	GAA ATG GCG CGT AAA ACA TTT
SaTlp2-3′	TTA TTT TCT TCT ACA GAT
TcpB-5′+Kozak	GCG ATG TCT AAA GAG AAA CAA GCC
TcpB-3′	TCA GAT AAG GGA ATG CAG T
**pIMAY cloning**
A	atatGGTACCGCTTGGAAATGATTTCTGAGTGTGGAATGG
B	CACAATTATATTCCACTGGTATGGAAATAATCGC
C	ATTTCCATACCAGTGGAATATAATTGTGTAAAAAACT
	ATAACTAAAATTGTAAGTCAATTTTAAC
D	atatGAGCTCTGTTGTCGTACAGTTTCTGCAGAATACC
OUT-F	TATTGCCATTTTCTCAAGATTCAAGTGG
OUT-R	TTACTCTCTCACCCTCTTGAAACTTTTCC

*Underlined: restriction sites.*

### COMPUTATIONAL MODELING OF SaTlp1

The secondary structure prediction and 3D template suggestions for structural SaTlp1, TcpB, and TLR2 were performed by HHpred (Soding et al., 2005) and used to generate a manually adjusted structural alignment. The PdTIR structure is comprised of four chains with differing structures which were manually aligned with an *Arabidopsis thaliana* TIR-like protein and human TIR domains from TLR2 (1FYW), TLR10 (1FYX), and MyD88 (2JS7) to the target sequences. The PDB structure 3LD8 was used to span the BB loop insertion which was not present in any of the TIR protein template sequences. MODELER version 9.10 (Fiser and Sali, 2003a) was used to generate 100 models and the poorly defined loop region (residues 43–49) of the highest ranking model refined by MODLOOP (Fiser and Sali, 2003b). Models were validated using PROCHECK (Morris et al., 1992), Verify3D (Eisenberg et al., 1997), and ProQ (Wallner et al., 2003). Sequence and structural similarity were analyzed by PDBeFold (Krissinel and Henrick, 2007).

### NF-κB LUCIFERASE ASSAY

The generation of HEK cells expressing TLR2, the main receptor for recognition of Gram-positive bacteria as well as the NF-κB reporter gene assay have been described recently (Willcocks et al., 2013). HEK cells expressing either boTLR2 or TLR5 (293-hTLR5; Invivogen) were seeded at a density of 2.5 × 10^5^ and were transfected after 24 h with 250 ng NF-kB-Luc (Firefly luciferase gene under the control of NF-κB promoter; Promega) and 1250 ng pSaTlp1-3.3, pSaTlp2-3.3, or pTcpB-3.3, using TurboFect^TM^ *in vitro* Transfection Reagent (Fermentas) or Lipofectamine 2000 (Invitrogen) following manufacturers’ guidelines and returned to the incubator for 24 h. A plasmid where *TcpB* had been ligated into pcDNA3.3 TOPO in the reverse orientation (pTcpB3.3^INV^) was used as a control. Cells were then split into six wells of a 24 well plate with three unstimulated wells and three wells of cells stimulated with either 100 ng ml^-1^ of the TLR2/6 ligand FSL-1 (Invivogen), 500 ng ml^-1^ of the TLR5 ligand flagellin (Invivogen) or 30 μg ml^-1^ heat-killed *E. coli*. (FBI Dummerstorf). After 24 h gene activation was analyzed using the Luciferase^®^ Reporter Assay System (Promega) following manufacturer’s guidelines. The cell lysates were made using passive lysis buffer, centrifuged at 16,000×*g* for 5 min and the OD 280 nm measured using a ND-1000 spectrophotometer (Nanodrop) to allow for normalization, as previously described (Liu et al., 2011).

### ASSESSMENT CYTOKINE PRODUCTION BY QUANTITATIVE PCR

To measure the effect of intracellular SaTlp1 or TcpB proteins on the production of inflammatory response genes in pbMEC, their mRNA concentration was determined for those cells transfected with the respective expression vector or the negative control plasmid. The pbMECs were grown to 80% confluence into 9 cm plates and were subsequently transfected with 1000 ng of the respective expression vector using Lipofectamine as described above. After an overnight recovery the transfected cells of each 9 cm plate were split into two wells of a six well plate (in order to obtain duplicates for the following inflammatory challenge). Subsequent to an additional recovery of 1 day the pbMEC were stimulated with 30 μg ml^-1^ heat-killed *E. coli* for three hours. After the stimulation total RNA was extracted using Trizol (Invitrogen) and RNA quality was determined as previously described (Gunther et al., 2011). For cDNA synthesis, 1.5 μg RNA was primed with a mixture of gene specific reverse oligonucleotide primers (**Table 3**) as described (Goldammer et al., 2004). After cDNA purification with HiPure columns (Roche) relative mRNA concentrations were determined with quantitative real-time PCR (RT-qPCR) using the LightCycler instrument and the SYBR green Plus kit (both from Roche), essentially as described previously (Goldammer et al., 2004; Gunther et al., 2011). Sequences of the oligonucleotide primers for the Chloride intracellular channel protein 1 (*CLIC1*; house-keeping gene), the proinflammatory cytokines *TNF-*α, *IL-1*β, *IL-6,* and *CXCL8*, inducible Nitric oxide synthase 2 (*iNOS2)*, the bactericidal β-defensin lingual antimicrobial peptide (LAP) and the acute phase protein Serum amyloid A (*SAA3)* are given in **Table 3**.

**Table 3:** Sequences of primer-pairs and probe used for qPCR reactions.

Gene Tested	Primer	Sequence
IL-1β	cDNA	5′-TGCCAGTCCTTGGGGTTATT
	Forward	5′-AACCGAGAAGTGGTGTTCTGC
	Reverse	5′-TTGGGGTAGACTTTGGGGTCT
IL-6	cDNA	5′-GGGAGCCCCAGCTACTTCAT
	Forward	5′-GGAGGAAAAGGACGGATGCT
	Reverse	5′-GGTCAGTGTTTGTGGCTGGA
CXCL8	cDNA	5′-GGCCCACTCTCAATAACTCTC
	Forward	5′-CCTCTTGTTCAATATGACTTCCA
	Reverse	5′-CATGGAACAATGTACATGCGAC
iNOS2	cDNA	5′-CCGGGGTCCTATGGTCAAA
	Forward	5′-ACAGGATGACCCCAAACGTC
	Reverse	5′-TCTGGTGAAGCGTGTCTTGG
LAP	cDNA	5′-TTTTTTTTTTTTTTTTTTTTN
	Forward	5′-AGGCTCCATCACCTGCTCCTT
	Reverse	5′-CCTGCAGCATTTTACTTGGGCT
SAA3	cDNA	5′-GCCAGCAGGTCTGAAGTGG
	Forward	5′-CTTTCCACGGGCATCATTTT
	Reverse	5′-CTTCGGGCAGCGTCATAGTT
TNFα	cDNA	5′-CTGTGAGTAGATGAGGTAAAGC
	Forward	5′-CTTCTGCCTGCTGCACTTCG
	Reverse	5′-GAGTTGATGTCGGCTACAACG
CLIC1	cDNA	5′-GATCCCCTCATCCTCAGCAC
	Forward	5′-AGAACAACCGCAGGTCGAAT
	Reverse	5′-GTCTCAGTCCGCCTCTTGGT

### ASSESSMENT OF CYTOKINE PRODUCTION BY ELISA

To measure the effect of intracellular SaTlp1 or TcpB proteins on the production of inflammatory response genes in pbMEC, the corresponding supernatants from pbMEC used to generate mRNA were analyzed for the presence of CXCL8 and TNF using ELISA systems as described recently (Metcalfe et al., 2014). IL-1β was analyzed using a bovine IL-1β ELISA kit (Pierce Protein Biology Products) as per manufacture’s recommendation.

### GENERATION OF *ΔsaTlp1/ΔsaTlp2* MUTANT

In order to delete *SaTlp1* and *SaTlp2* from the ST398 genome the pIMAY plasmid and protocols were used as previously described (Monk et al., 2012). Deletion constructs were produced by amplifying regions 500-bp upstream and downstream of the genes using primer pairs A/B and C/D respectively. These PCR products were used as templates for the spliced overlap extension (SOE) PCR using A/D primers. The PCR product was purified and cleaved at endonuclease sites added by the A and D primers and cloned into pIMAY using standard protocols. Allelic exchange was achieved by transforming electrocompetent ST398 with the pIMAY construct and plating on brain heart infusion agar (BHIA) with chloramphenicol (Cm) at 28° C (Monk et al., 2012). Potential mutants were identified after antisense *secY* induction by colony PCR with OUT-F/OUT-R primers. The double mutant was confirmed by sequencing the deleted region from isolated genomic DNA.

### MURINE INFECTION MODEL

The murine peritoneal *S. aureus* infection is generally considered by be a good model to assess invasiveness and subsequently infectivity of *S. aureus*. The study was performed at the AAALAC-accredited Michigan State University *In vivo* Facility (East Lansing, MI, USA, study number 14IVF-IF-1003, IACUC approval No. 01/13-013-00^4^). The study was conducted in accordance with the current guidelines for animal welfare (Guide for the Care and Use of Laboratory Animals, 8th Edn, 2011). The procedures used in this study were reviewed and approved by the Institutional Animal Care and Use Committee. Six- to eight-week-old female CD-1 mice (Charles River, Portage, MI, USA) with an average weight of 23.8 gm were randomly assigned into 13 groups (*n* = 6 per group). Animals were housed three per cage and provided food and water *ad libitum*. Animals were acclimated 8 days prior to use. One day prior to infection a parent culture of *S. aureus* ST398, and the KO strain *ΔSaTlp1/ΔSaTlp2* were removed from frozen storage and streaked out onto trypticase soy agar supplemented with 5% lysed sheep blood cells (TSA-5% SB). Cultures were grown overnight at 37°C, at ambient atmosphere. On the day of infection, the cultures were removed from the incubator and used to prepare an inoculum of each organism in trypticase soy broth (TSB). Bacterial concentrations were measured in the broth by forward light scatter measurement at 600 nm (O.D.600) and adjusted to provide a target value of 0.600. A 1:100 dilution was prepared, using TSB supplemented with 10% gastric hog mucin (GHM) as the diluent, to generate a high dose concentration for challenge of 7.0 log10 bacteria in a dose volume of 0.5 mL. Serial 1:10 dilutions of the high concentration were made into TSB-10% GHM to prepare 6.0 log10, 5.0 log10, 4.0 log10, 3.0 log10, and 2.0 log10 challenge doses of each strain, and the inoculum concentration of each dosing preparation was confirmed by plating serial dilutions for CFU enumeration. Animals received mucin control, wild-type *S. aureus*, or KO *S. aureus* as a single intraperitoneal injections. Animals were monitored at least twice daily for signs of morbidity. During periods of expected high mortality, mice were monitored a minimum of four times per day. Animals exhibiting clinical signs listed as criteria for implementation of early euthanasia in AUF 01/13-013-00 were euthanized by CO2 asphyxiation, followed by cervical dislocation. Deaths were recorded as the number “died” or “euthanized” on a daily basis, and the study was terminated on Day 6. Any remaining animals were euthanized at that time.

### STATISTICAL ANALYSIS

All *in vitro* experiments were performed at least three times, with samples run in at least duplicates. Data are presented as averages of all experiments, and results were assessed for statistical significance by a two-way ANOVA followed by a Bonferroni *t*-test between treatment groups using Excel and GraphPad Prism software packages (version 5; GraphPad Software, Inc., La Jolla, CA, USA).

For the *in vivo* experiment, data were collected on an Excel spreadsheet. Survival curves were compared statistically using Log-rank (Mantel-Cox) and Gehan-Breslow-Wilcoxson tests (GraphPad Prism 5.03). Differences were considered significant at *p* < 0.05. LD50 curves were generated using GraphPad Prism 5.03. A sigmoidal Emax dose response model derived from the Hill equation (four-parameter logistic equation with variable slope using GraphPad Prism v5.03) was used to determine the relationship between the antibacterial drug dose and the efficacy, as determined by bacterial burden: Y = D +(A-D)/(1 +10(log C–X)*α). For the four-parameter logistic equation, *Y* is the observed effect, *D* is the bottom, *A* is the top, *C* is the LD50 or 50% of the observed maximum effect, *X* is the log of the challenge concentration and α is the Hill slope. The bottom value (D) was constrained to 0 and the top value (C) was left unconstrained. Data are shown as Kaplan–Meier Survival Curves.

## RESULTS

### SaTlp1 AND SaTlp2 SHARE STRUCTURAL HOMOLOGY TO KNOWN TIR DOMAINS

**FIGURE 1:**
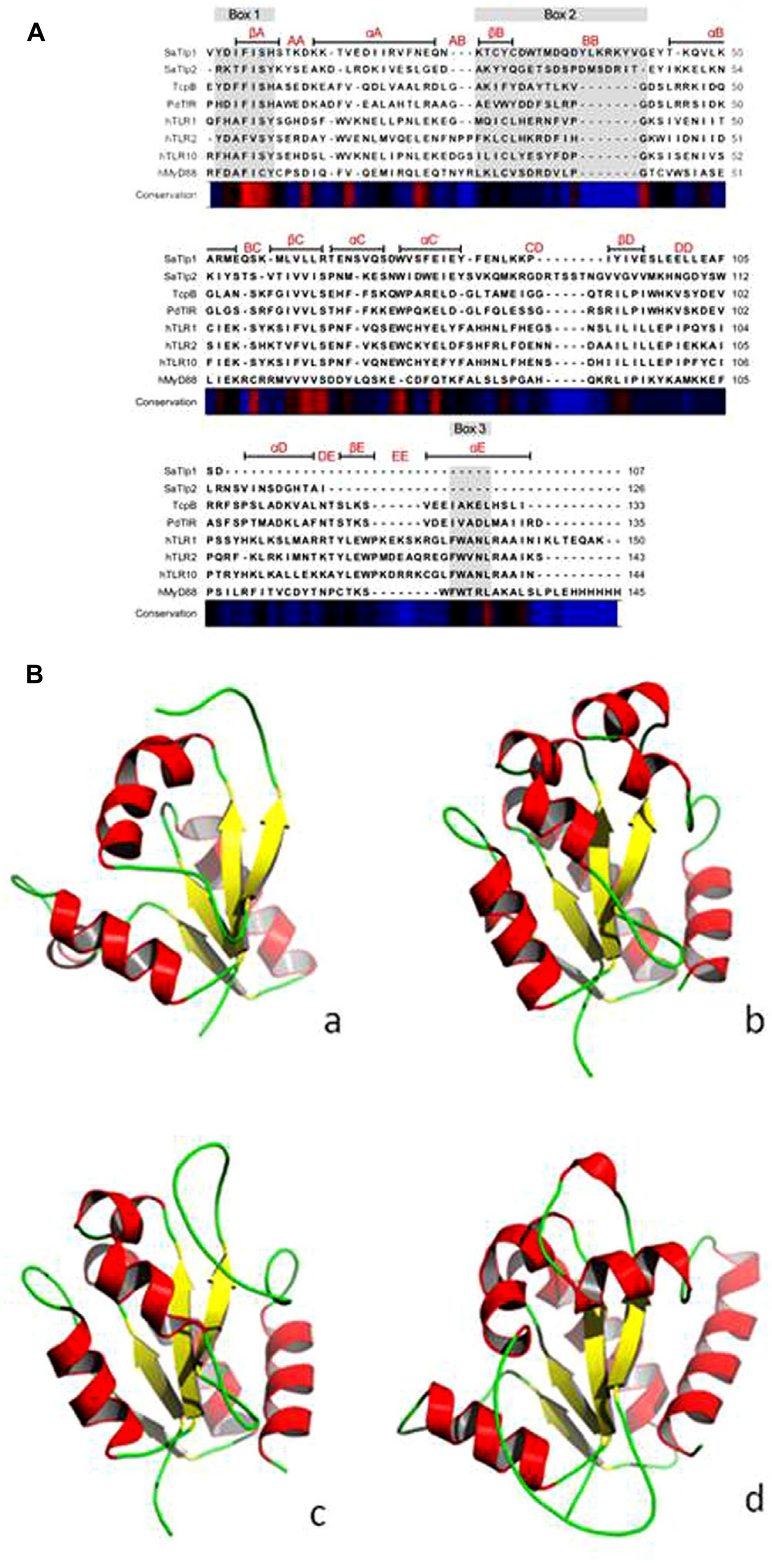
**(A)** Alignment of the DUF1863 domains of SaTlp1, SaTlp2, and TcpB with TIR domains of known structure. The regions corresponding to the TIR motifs boxes 1, 2, and 3 are indicated by the rectangles above the alignment, highlighted in gray. Secondary structure elements corresponding to the known proteins are annotated above with conservation from high (red) to low (blue) shown below. **(B)** Predicted tertiary structure of (a) SaTlp1, (b) TcpB and (d) boTLR2 and crystal structure of (c) PdTIR.

Initially we tried to identify Tcp in *S. aureus* using a bioinformatics approach. As Tcps in other bacteria have been shown to show similarities to mammalian TLR TIR domains, we compared different TLR TIR domains against all available *S. aureus* genomes (taxid:1280) using BLAST. This resulted in the identification of two immediately adjacent proteins (*e* = 8 × 10^-6^ and *e* = 3 × 10^-5^, respectively) containing a DUF1863 domain, located within a recently identified putative transposon. SMART annotation confirmed that these proteins, named *S. aureus* Tcp-like protein 1 and 2 (SaTlp1 and SaTlp2) contain DUF1863 domains_._ SaTlp1 and SaTlp2 share less than 20% sequence identity with other members of the TIR superfamily. However, despite low similarity at the sequence level, secondary structure prediction suggests that at least four of the central beta strands common to TIR-like domains are present in these bacterial proteins. The closest structural homologs identified by HHpred are matches to the DUF1863 (PF08937) and TIR or TIR_2 (PF13676 and PF01582) domain-containing families and proteins sharing a Flavodoxin-like fold (SCOP 31128 and 31130). The closest secondary structure match to both SaTlp1 and TcpB was PdTIR (PDB: 3H16) while the *Eubacterium rectale* putative signal transduction protein containing a Flavodoxin-like fold (PDB: 3HYN) was the top ranked hit for SaTlp2 with probabilities of 99.9, 100, and 99.7%, respectively. Within eukaryotic TIR domains, three conserved regions called boxes 1, 2, and 3 are proposed as putative adaptor binding sites, essential for signaling. The box 1 motif can be clearly identified in all of the TIR-like domains included in the alignment (**Figure 1A**). However, both SaTlp1 and SaTlp2 appear to have a helical insertion within the BB loop (box 2) and, unlike the other bacterial proteins shown, seem to entirely lack box 3. Computational modeling of SaTlp1 predicts the conserved TIR-like tertiary structure of four/five central beta strands surrounded by helices (**Figure 1B**). While the modeled structure of TcpB is most similar to PdTIR (∼0.5 Å), SaTlp1 shows closer structural homology to TLR TIR domains (∼1.9 Å).

### *SaTlp1* AND *SaTlp2* ARE PRESENT IN ALL ST398 ISOLATES AND CC75 *Staphyloccoccus aureus*

**FIGURE 2:**
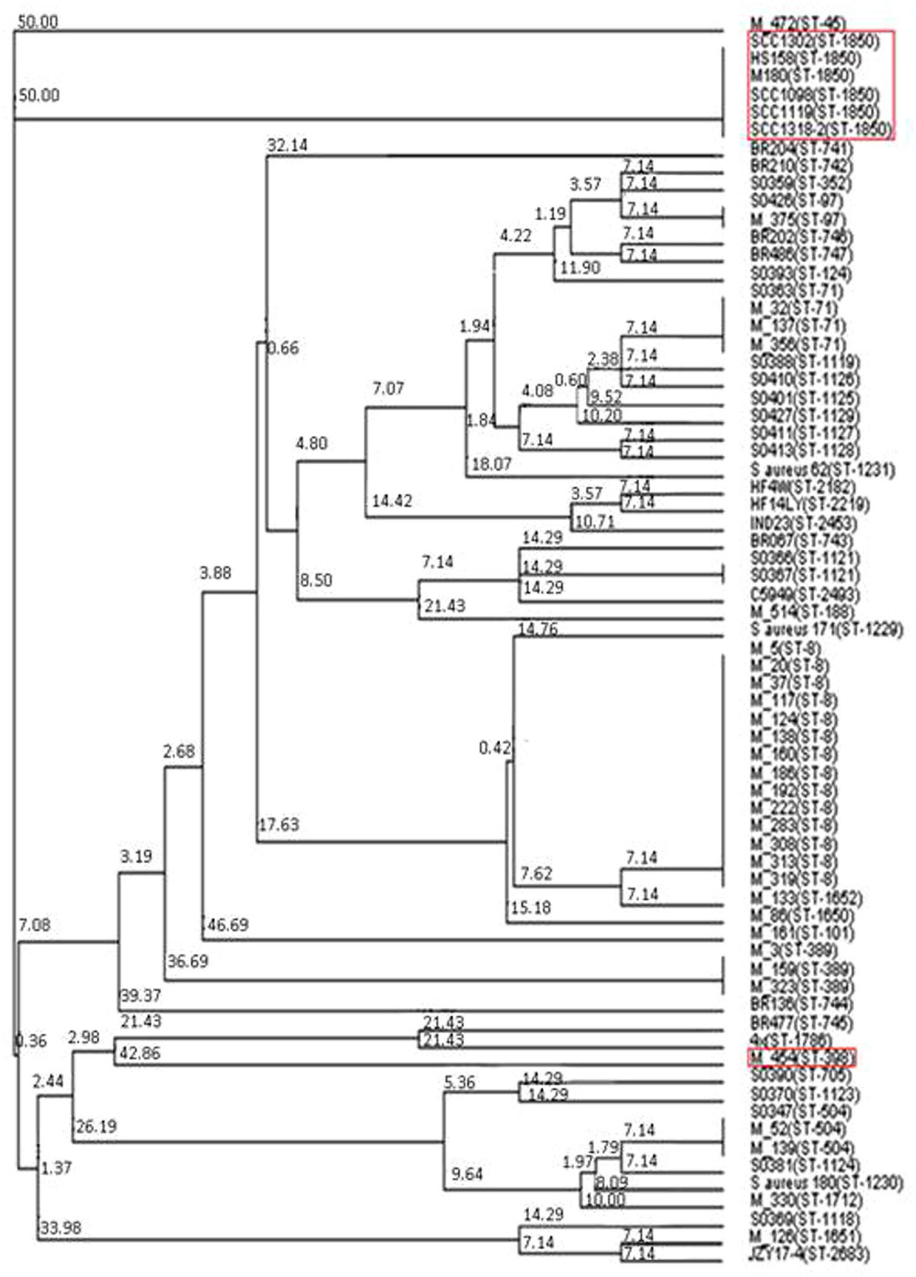
**Neighbor joining tree of *S. aureus* isolates.** Strains falling under “Bovine Mastitis” disease classification on MLST.net and ST398 and ST1850. Red boxes indicate STs in which SaTlp1 and SaTlp2 have been identified. The tree was computed by using concatenated nucleotide sequences of seven housekeeping genes and was constructed using the tool provided by the MLST database (http://saureus.mlst.net/sql/uniquetree.asp?). Bootstrap values are indicated above the branches.

Having identified two putative Tcp-like proteins, we next assessed the occurrence of these genes in different *S. aureus* strains. To do so, a phylogenetic tree of all *S. aureus* strains used in this study was created by MLST analysis, and the presence of *SaTlp1* and *SaTlp2* in these strains determined (**Table 1**). Interestingly, *SaTlp1* and *SaTlp2* were identified in all ST398 isolates tested, regardless of the species of isolation, clinical symptoms of the host-species or methicillin-sensitivity status. Additionally, BLAST searches on 89 ST398 isolates were performed which revealed that all of these contained these genes. In addition to ST398, BLAST searches revealed these genes were present in *S. aureus* clonal complex (CC) 75 (also known as *S. argenteus*), however, they were not detected in any other *S. aureus* lineage. ST398 and CC75 are only distantly related (**Figure 2**) and published work on CC75 states that it is a highly divergent lineage, showing an average seven fold greater nucleotide divergence in orthologous genes with typical *S. aureus* strains than other strains (Holt et al., 2011). Considering this divergent relationship but the similarity of these genes it seems highly likely that the transposon containing them was transferred between the strains post-dating their divergence.

### BOTH SaTlp1 AND SaTlp2 UP-REGULATE NF-κB ACTIVATION IN HEK-boTLR2 AND pbMEC

**FIGURE 3:**
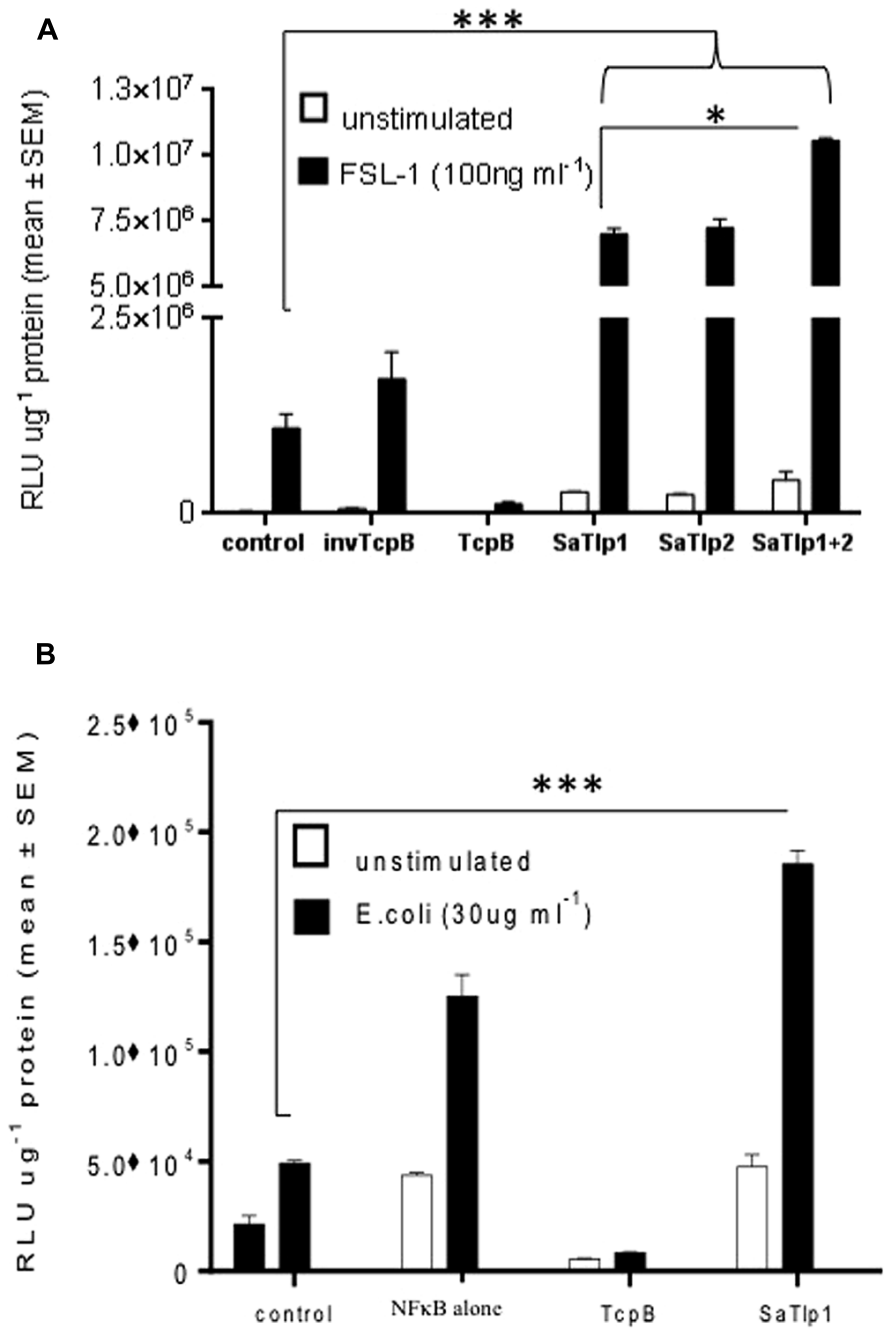
**Impact of SaTlp1 and SaTlp2 on NF-κB activation in two different cell types.** Cells were transfected as described, and relative luciferase units were analyzed. Graphs showing the RLU per μg protein in lysates from **(A)** HEK293-boTLR2 cells stimulated with 100 ng ml^-1^ FSL-1 and **(B)** primary bovine mammary epithelial cells (pbMECs) stimulated with 30 μg ml^-1^ heat-killed *E. coli.* Cells were transfected with plasmids indicated. Error bars represent the standard error of the mean for each condition (±SEM) of three independent repeats performed in triplicates. Control: medium alone; NFkB alone: cells transfected with NFkB coding plasmid only; invTcpB: cells transfected with a plasmid in which the TcpB sequence was inserted in reverse direct; SaTlp1: cels transfected with plasmid coding for SaTlp1; SaTlp2: cells transfected with plasmid coding for SaTlp2; SaTlp1+2: cells transfected with both plasmids. Significant fold changes are denoted by asterisks in the figure (*p < 0.05; ***p < 0.001).

To assess whether the presence of the Tcp-like proteins in this *S. aureus* strain interferes with the activation of the innate immune response, we tested whether the identified proteins would exhibit similar effects as described for Tcps of other bacteria which seem to inhibit TLR signaling. We analyzed whether transfection of HEK cells expressing boTLR2 with SaTlp1 and SaTlp2 affected the downstream activity of the transcription factor NF-κB after stimulation with the TLR2 ligand FSL-1 (**Figure 3A**). The levels of NF-κB-induced luciferase were significantly up-regulated following transfection with *SaTlp1* and *SaTlp2* plasmids (both *p* < 0.001) when compared to the negative control. Simultaneous transfection of both plasmids also up-regulated NF-κB activation (*p* < 0.001). In the HEK-boTLR2 system these plasmids up-regulated NF-κB-dependent luciferase between 4.1 (*SaTlp1*) and 6.1 (*SaTlp1/2*) fold following stimulation compared to the negative control. In contrast, transfection of HEK-boTLR2 cells with *TcpB* which had been shown before to block TLR-dependent NF-κB activation (Radhakrishnan et al., 2009) did not induce significant NF-κB stimulation, confirming these data. FSL-1 failed to activate NF-κB in HEK293 not expressing TLR2 (Data not shown).

As the HEK cell system is a somewhat artificial system, we next assessed the effects of SaTlp1 using pbMECs which express TLR2 and have been used before to assess the effect of mastitis causing pathogens on the innate immune response (Yang et al., 2008; Gunther et al., 2011). As seen in HEK293 cells, transfection of *SaTlp1* (*p* = 0.006) caused increased NF-κB activation in pbMEC in response to heat-killed *E. coli*. As before transfection of pbMECs with TcpB before *E. coli* stimulation did not activate NF-κB signaling (*p* < 0.001). However, this up-regulation was overall lower compared to HEK293 cells with *SaTlp1* up-regulating NF-κB by 1.4 fold compared to the negative control (**Figure 3B**).

### THE EFFECT OF SaTlp1 AND SaTlp2 IS NOT LIMITED TO ONE SPECIES OR A SPECIFIC TLR

**FIGURE 4:**
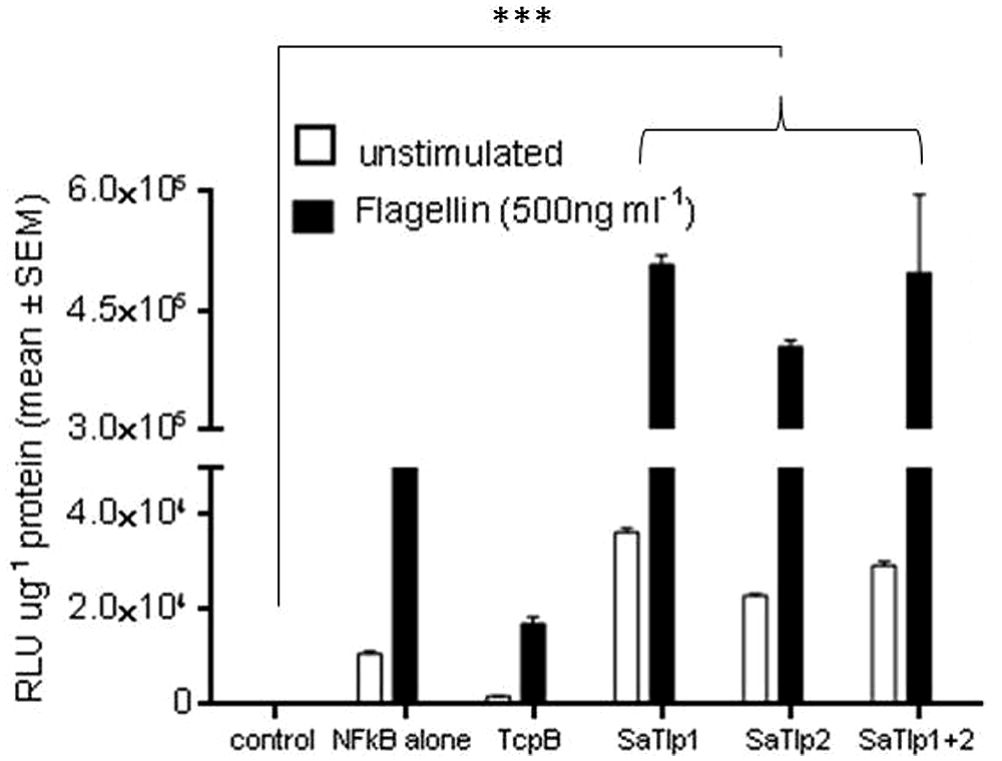
**Impact of SaTlp1 and SaTlp2 on NF-κB activation in HEK293-hTLR5 cells.** Graphs showing the RLU per μg protein in lysates of cells stimulated with stimulated with 500 ng ml^-1^ flagellin. Cells were transfected with plasmids indicated. Control: medium alone; NFkB alone: cells transfected with NFkB coding plasmid only; SaTlp1: cells transfected with plasmid coding for SaTlp1; SaTlp2: cells transfected with plasmid coding for SaTlp2; SaTlp1+2: cells transfected with both plasmids. Error bars represent the standard error of the mean for each condition (±SEM) of three independent repeats performed in triplicates. Significant fold changes are denoted by asterisks in the figure (***p < 0.001).

To assess whether the responses seen in both cell types would be unique to the interaction with boTLR2, we examined the effect of SaTlp1 and SaTlp2 in HEK cells transfected with hTLR5 cells stimulated with flagellin (**Figure 4**). Transfection of *SaTlp1 SaTlp2* (both *p* < 0.001) and *SaTlp1/2* (*p* < 0.001) led to an up-regulation of NF-κB activity of between 2.0 (*SaTlp2*) and 2.5 (*SaTlp1*) fold. In contrast, transfection of HEK cells expressing hTLR5 with *TcpB* down-regulated its activation in response to flagellin (*p* < 0.001). These results are therefore similar to those for boTLR2 expressing cells, suggesting the action of both proteins may not be limited to a specific TLR.

### UP-REGULATION OF NF-κB ACTIVATION BY SaTlp1 INCREASES mRNA AND PROTEIN EXPRESSION OF PRIMARY AND SECONDARY RESPONSE GENES

**FIGURE 5:**
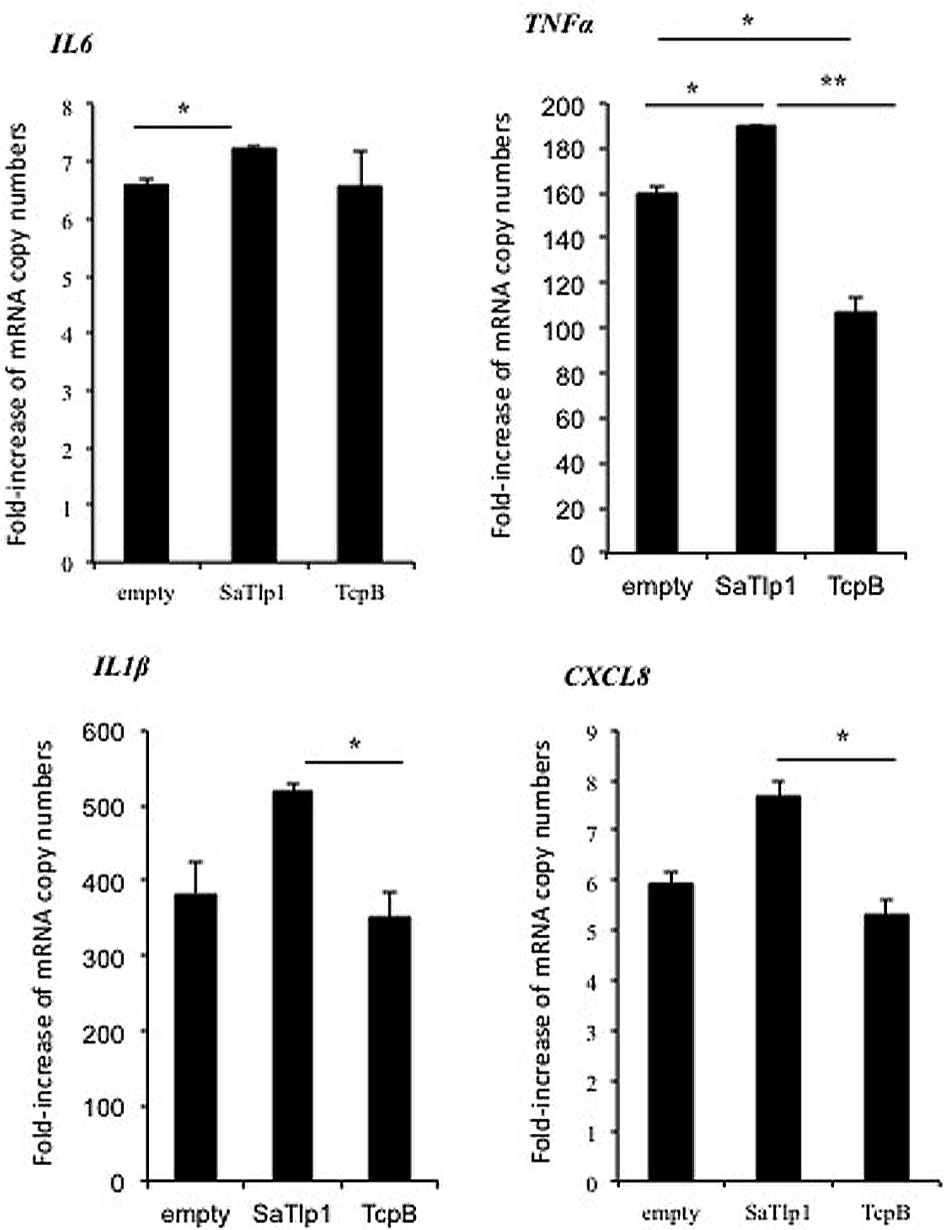
**Average fold increase of mRNA levels of the pro-inflammatory genes *IL-1*β, *IL-6, TNF, CXCL8* in pbMEC transfected with the empty plasmid, or plasmids coding for TcpB and SaTlp1 and stimulated with 30 μg ml^**–1**^ heat-killed *E. coli* for three hours.** Error bars represent the standard error of the mean for each condition (±SEM) of three independent repeats performed in triplicates. Significant fold changes are denoted by asterisks in the figure (*p < 0.05; **p < 0.01).

**FIGURE 6:**
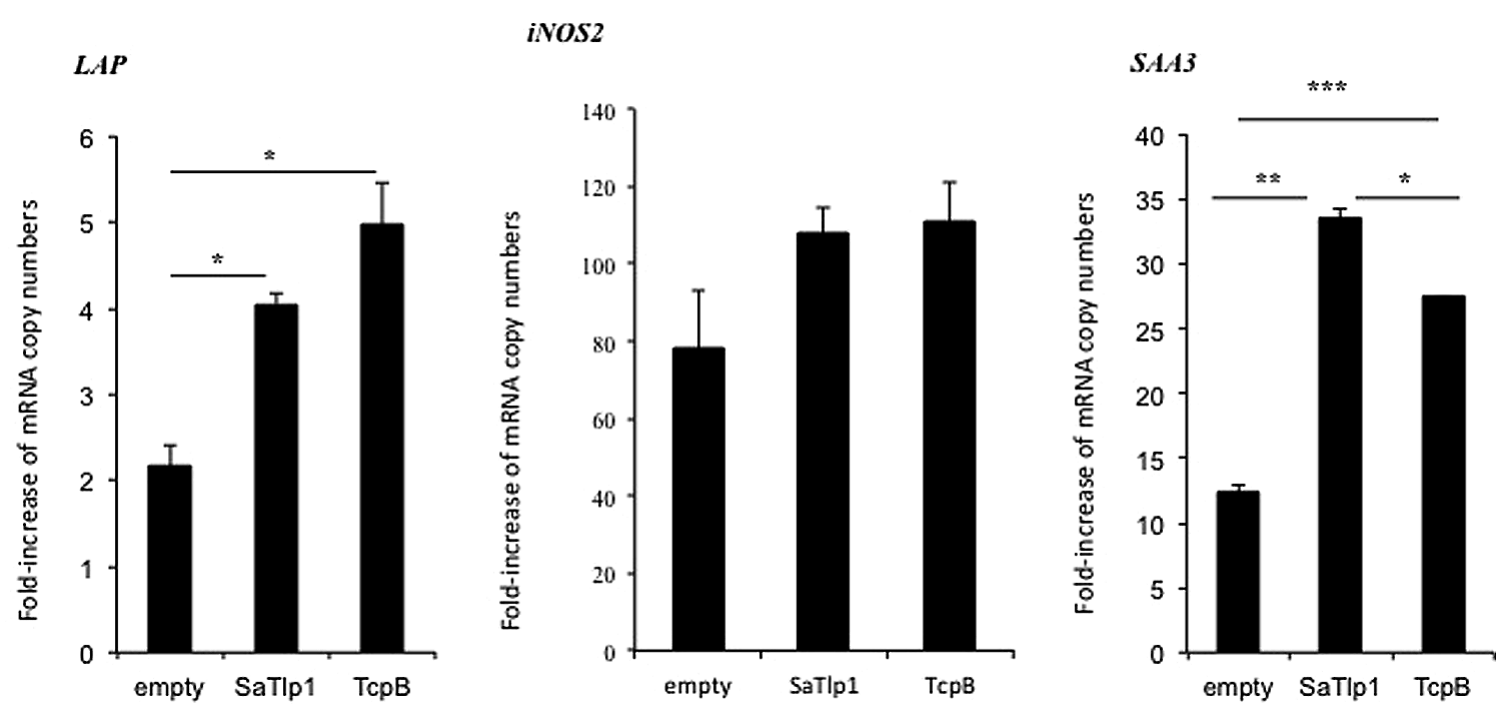
**Average fold increase of mRNA levels of the secondary response genes *iNOS-2 LAP* and *SAA3* in pbMEC transfected with the empty plasmid or plasmids coding for TcpB and SaTlp1 and stimulated with 30 μg ml^**–1**^ heat-killed *E. coli* for three hours.** Error bars represent the standard error of the mean for each condition (±SEM) of three independent repeats performed in triplicates. Significant fold changes are denoted by asterisks in the figure (*p < 0.05; **p < 0.01; ***p < 0.001).

Having established that SaTlp1 stimulated NF-κB activation, we investigated whether this was also reflected at the transcriptional level for NF-κB-dependent cytokine production. We analyzed expression of the primary response genes *IL-1β*, *IL-6*, *CXCL8,* and *TNFα* as well as the secondary response genes *iNOS-2*, *LAP,* and *SAA3* by qPCR, similar to that described before (Gunther et al., 2011). Stimulation of pbMEC transfected with either empty plasmids or plasmids containing *SaTlp1* followed by *E. coli* stimulation resulted in the up-regulation of *IL-1*β, *IL-6*, *CXCL8,* and *TNF-*α mRNA, levels to various extents (**Figure 5**). In contrast, transfection of pbMEC with a plasmid coding for TcpB in general reduced expression of these genes after *E. coli* stimulation (**Figure 5**). Transfection of pbMEC with plasmids coding for SaTlp1 and TcpB followed by subsequent stimulation with *E. coli* increased mRNA expression for the secondary response genes *iNOS-2*, *LAP* and *SAA3* (**Figure 6**), with *LAP* expression being effected more by TcpB.

**FIGURE 7:**
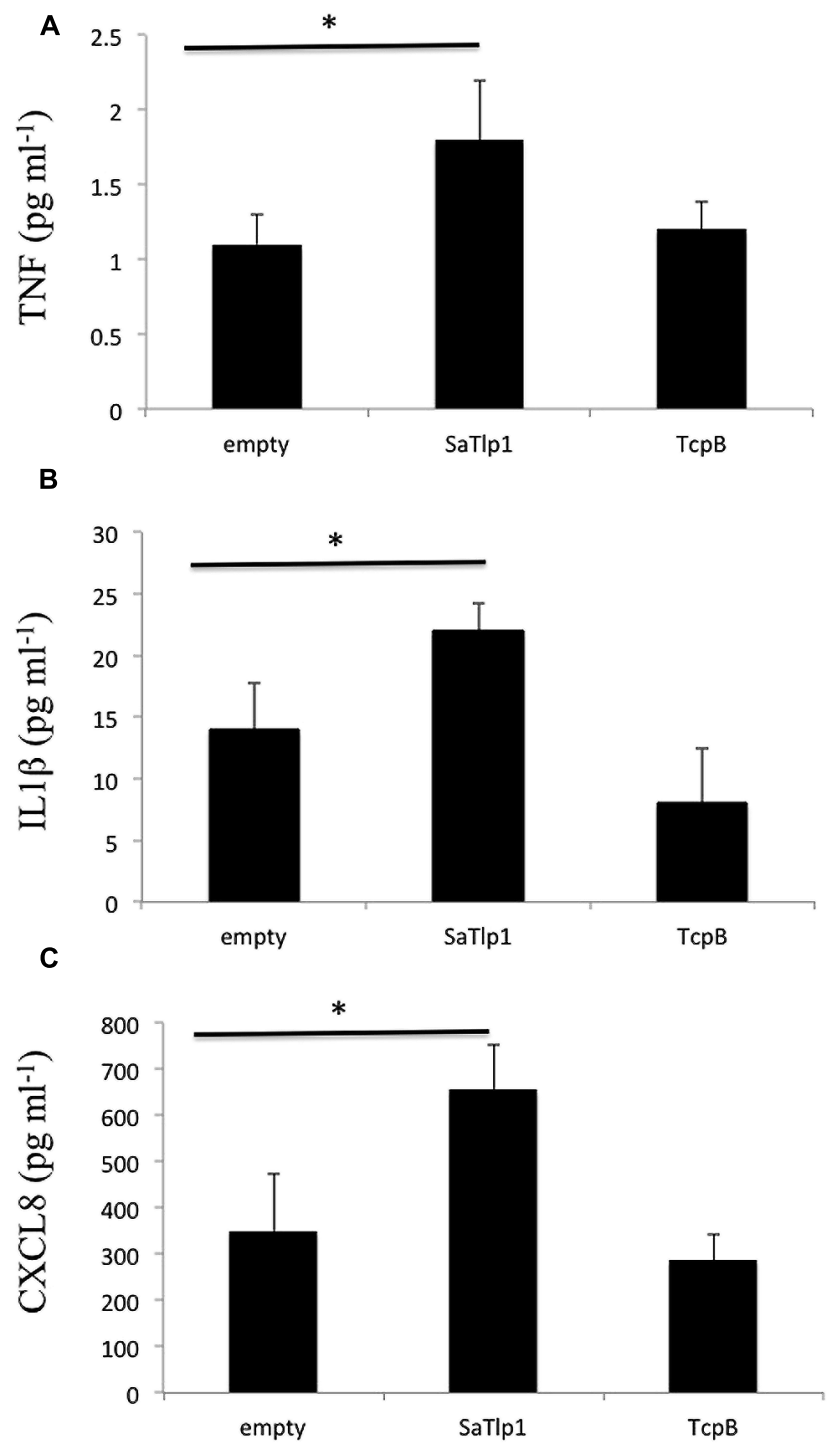
**Values of the pro-inflammatory TNF **(A)**, IL-1β **(B)**, and CXCL8 **(C)** analyzed in the supernatants of pbMEC transfected with the empty plasmid, or plasmids coding for TcpB and SaTlp1 and stimulated with 30 μg ml^**–1**^ heat-killed *E. coli* for three hours.** Error bars represent the standard error of the mean for each condition (±SEM) of three independent repeats performed in triplicates. Significant fold changes are denoted by asterisks in the figure (*p < 0.05).

Corresponding supernatants were analyzed for the presence of CXCL8, TNF-α, and IL-1β. The secretion pattern followed in general the results obtained for qPCR, with values obtained for all three cytokines being in generally low. Similar as to the data obtained by qPCR, supernatants of pbMEC transfected with SaTlp1 before stimulation with *E. coli* contained the largest amount of all three cytokines (**Figures 7A–C**), whereas no differences were seen between untransfected pbMEC and pbMEC transfected with TcpB.

### A ΔsaTlp1/ΔsaTlp2 ST398 POTENTIALLY IMPACTS ON SURVIVAL RATE IN A *Staphylococcus aureus* MICE CHALLENGE MODEL

**FIGURE 8:**
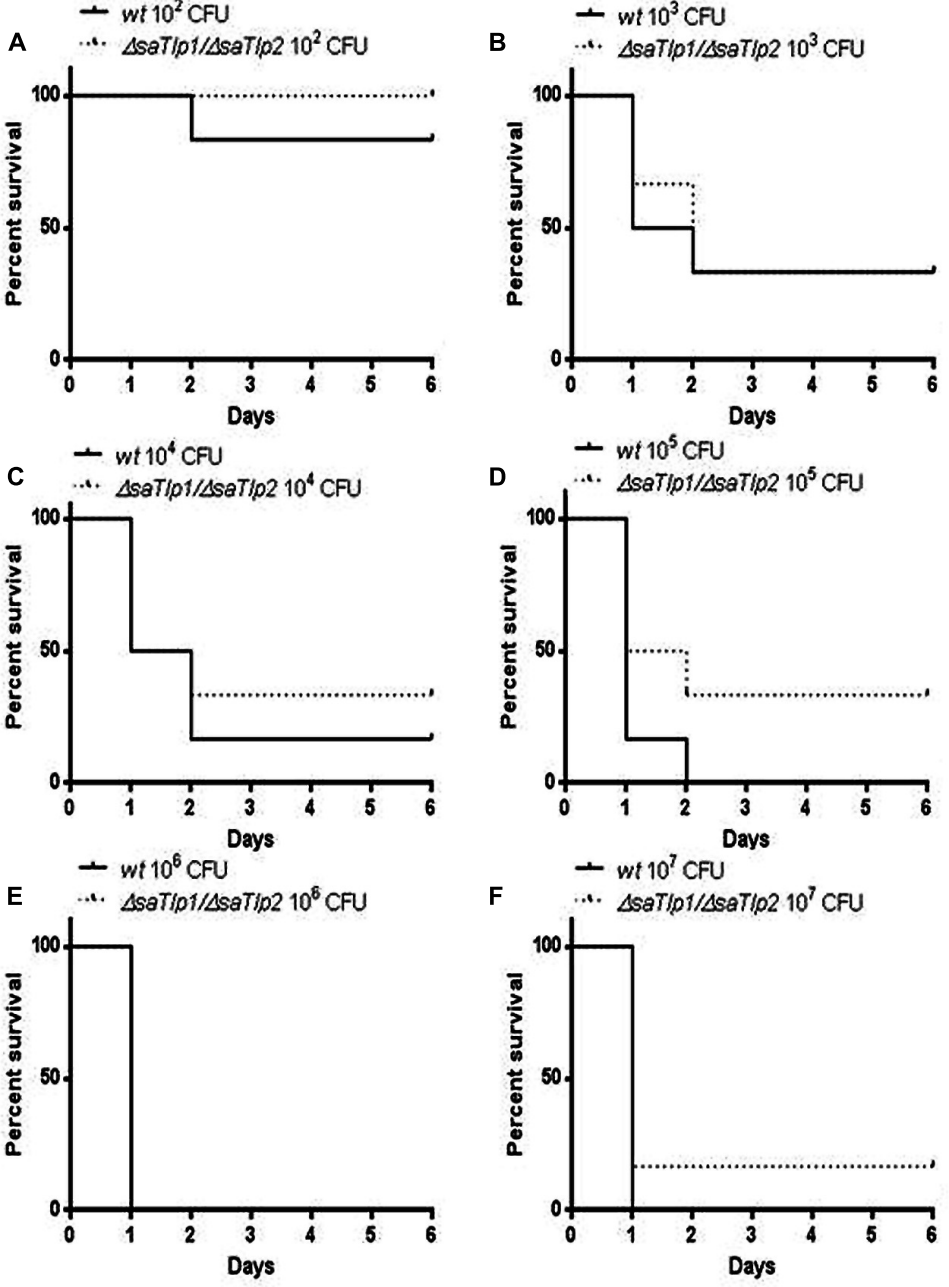
**Kaplan–Meier survival curves for groups of mice infected with different CFU of either wild-type or *ΔsaTlp1/ΔsaTlp2* ST398 *S. aureus*.** Each figure represent six mice challenged by intra-peritoneal injection with 0.5 mL containing wild-type ST398 and six mice challenged with *ΔsaTlp1/ΔsaTlp2* KO mutant which were then monitored for 6 days and deaths recorded. Figures show survival rates for mice challenged with **(A)** 10^2^ CFU **(B)** 10^3^ CFU **(C)** 10^4^ CFU **(D)** 10^5^ CFU **(E)** 10^6^ CFU and **(F)** 10^7^ CFU.

As the presence of SaTlp1 and SaTlp2 seemed to increase NF-κB activation and pro-inflammatory cytokine production in response to subsequent stimulation, we next assessed whether the absence of the genes has an impact on the virulence of *S. aureus* ST398 *in vivo*. After creation of a *ΔsaTlp1/ΔsaTlp2* ST398 KO mutant, the growth rate or colony appearance of both wild-type and mutant *S. aureus* were compared and showed no difference (data not shown). In order to determine whether there are differences in the *in vivo* pathogenicity of wild-type and *ΔsaTlp1/ΔsaTlp2* ST398 a murine sepsis model was used in order to calculate the median lethal dose (MLD) of the bacteria. CD-1 mice were challenged via i.p. injection with 10^2^–10^7^ CFU of either wild-type or mutant *S. aureus* after which the time until their death was monitored. The data showed a trend for increased survival rates for mice infected with the *ΔsaTlp1/ΔsaTlp2* mutant, most noticeably in the case of infection with 10^5^ CFU when 2 of 6 mice challenged with mutant bacteria survived until day 6 of the experiment whereas all mice infected with wild-type had died by day 2 (**Figure 8**). Despite the fact that none of the observed differences reach level of significance, the lowest *p*-value (*p* = 0.13) was obtained for 10^5^ CFU. Considering the data from all challenge concentrations, the MLD of the strains was calculated using the Reed-Meunch equation. This showed the MLD of *ΔsaTlp1/ΔsaTlp2* ST398 to be 3630 CFU compared to one of 575 for the wild-type ST398 indicating that in order for 50% of challenged mice to be killed by the mutant, an infectious dose of over 6.3 times that of wild-type ST398 is required. This suggests that although differences between wild-type and *ΔsaTlp1/ΔsaTlp2* ST398 are not significant at individual concentrations, when is considered together there does appear to be genuine differences in their pathogenicity.

## DISCUSSION

Staphylococci have evolved numerous mechanisms to evade immune recognition (Foster, 2005). In the present study, we describe the identification of two DUF1863 domain-containing proteins in the emerging zoonotic *S. aureus* ST398 which show structural similarity to TLR TIR domains. As described for other proteins of the STIR-superfamily, both proteins show only a weak sequence homology, with the mentioned boxes 1 and 2 being identifiable but not box 3. Furthermore our data suggests that expression of these proteins results in significantly increased activity of the transcription factor NF-κB in HEK-boTLR2, HEK-hTLR5, and pbMEC cells, as well as mRNA expression levels of pro-inflammatory primary and secondary response genes. However, in contrast to work published on the function of Tcps identified in Gram-negative bacteria, which were shown to down-regulate NF-κB activation as well as subverting the ensuing innate immune response (Radhakrishnan et al., 2009; Yadav et al., 2010; Rana et al., 2011), our data seems to suggest that these proteins activate NF-κB signaling and the production of inflammatory mediators. Although there is still a certain amount of debate as to whether TIR, SEFIR, and DUF1863 domains should be classified separately or as a single domain, our data suggests that they may have distinct functions within the same signaling pathways.

In addition to the differences seen in activation of NF-κB, this study is the first to investigate these presence of such proteins from Gram-positive bacteria, as all published work on bacterial Tcps has been in Gram-negative bacteria such and *B. melitensis* (TcpB), *E. coli* (TcpC), and *P. denitrificans* (PdTIR). It is unclear whether NF-κB down-regulation is associated with Gram-negative and up-regulation with Gram-positive bacteria or if there are examples of up- and down-regulation for Tcp present in both. Further to this it must be considered that TIR domains are most likely to have more general protein/protein interaction functions, as reviewed recently (Spear et al., 2009), and modulation of TLR signaling by these proteins may not be the rule.

Although the identified proteins are likely to be ubiquitous within ST398, they appear to be rare within the *S. aureus* species as a whole. A recently published genome, however, showed that they are present in a very distantly related, early branching *S. aureus* strain CC75, isolated in Northern Australia, also known a *S. argenteus* (Holt et al., 2011). There is conflicting evidence as to whether the genetic element containing these genes is ancestral or acquired and further study would determine if there are any notable pathogenic similarities between these two strains, particularly in the context of ST398. Strains of this group are notable for their high promiscuity, with the ability to easily cross species-barriers, having been isolated from a range of livestock animals and also humans. Furthermore ST398 *S. aureus* are often missing several traditional staphylococcal virulence factors such as PVL, suggesting that these bacteria may have acquired alternative factors that allow for their successful host colonization. It could be hypothesized that these Tlps may represent novel staphylococcal virulence factors, which may be unique to this specific MLST. Indeed, and in contrast to our data, the interference of *S. aureus* with MyD88-dependent TLR signaling was recently suggested (Gunther et al., 2011), with the authors demonstrating an inhibitory effect of *S. aureus* on TNF-α and IL-1 mRNA production. The fact that our data show that the identified proteins are influencing the pro-inflammatory response system on both, mRNA and protein level indicates that both proteins are potentially interesting virulence factors that warrant further investigation. This is of even more interest as not only SaTlp1, but also TcpB from *B. melitiensis* seemed to affect mRNA expression levels of the secondary response genes *LAP* and *SAA3*. These require *de novo* synthesis and have been shown to need chromatin-remodeling (Liu et al., 2011) or the transcription factor C/EBPδ (Litvak et al., 2009), and were differently expressed compared to the pro-inflammatory genes.

We are fully aware that the *in vivo* model used may not be the most appropriate one to assess the function of the two identified *S. aureus* proteins. However, murine models for *S. aureus* are still considered the appropriate standard for invasiveness/survival (Kim et al., 2014; Zhang et al., 2014). The *in vivo* infection data from a murine sepsis model provided data which suggests a trend toward increased survival time for mice infected with the *ΔsaTlp1/ΔsaTlp2* mutant compared to the wild-type ST398 strain although differences between these were not statistically significant at any individual *S. aureus* concentration. The level where the greatest differences are apparent was when mice were infected with 10^5^ CFU. This is with a *p*-value of 0.1292 according to the Log-rank test which, although not significant, would certainly merit further investigation considering the relatively small sample size at each CFU concentration (*n* = 6). It is possible that at CFU concentrations lower than 10^5^ there may be less effect as the mice may be more able to fight the infection regardless, whereas at higher concentrations the effects of these proteins may be negated due to the sheer number of bacteria present. Around the 10^5^ mark may represent something of a sweet spot where effect of these proteins can be seen. Additionally the small sample size is clearly susceptible to natural variation between mice and challenge doses. This is especially important as while large differences in survival of infected mice were not observed there may be more subtle *in vivo* effects of these proteins similar to those proposed for YpTlp (Spear et al., 2012). Subtle *in vivo* effects would very likely require relatively large sample sizes in order to be able to produce statistically significant results. The exact mechanisms used by these proteins are yet to be elucidated, as a direct interaction with host proteins has not been identified. It should also be noted that there is still no clear picture as to how these bacterial proteins would reach any cellular target, as they do not contain domains associated with export from the bacterium. However, these findings are similar to TcpB of *Brucella*. Despite this, TcpB exhibits lipid-binding properties (Radhakrishnan and Splitter, 2010), and interacts with phosphoinositides through its N-terminal domain, leading to co-localization with the plasma membrane and components of the cytoskeleton (Radhakrishnan et al., 2009). Furthermore this *Brucella* TIR protein has the property of cell permeability which would provide a mechanism for virulence and could mean that it influences nearby cells not infected with the bacteria, as well as contributing to trafficking within the cell to influence host cell machinery.

In conclusion, SaTlp1 and SaTlp2 are potential novel virulence factors in ST398 *S. aureus* which appear to interact with the innate immune signaling machinery of host cells in novel ways. The presence of these proteins may account for some of the unusual characteristics of strains of *S. aureus* within ST398 and are therefore interesting targets for understanding these characteristics.

## Conflict of Interest Statement

The authors declare that the research was conducted in the absence of any commercial or financial relationships that could be construed as a potential conflict of interest.

## Footnotes

1 http://blast.ncbi.nlm.nih.gov

2 http://smart.embl-heidelberg.de/

3 http://bioinfo.pl/

4 http://www.aaalac.org/accreditedorgsdirectorysearch/aaalacprgms.cfm
